# Prevalence, incidence, and medications of narcolepsy in Japan: a descriptive observational study using a health insurance claims database

**DOI:** 10.1007/s41105-022-00406-4

**Published:** 2022-08-30

**Authors:** Aya Imanishi, Yuta Kamada, Kai Shibata, Yukinori Sakata, Hiroaki Munakata, Mika Ishii

**Affiliations:** 1 Department of Neuropsychiatry, Akita University School of Medicine, Akita, Japan; 2 Clinical Planning and Development Department, Medical HQs, Eisai Co., Ltd, Tokyo, Japan

**Keywords:** Narcolepsy, Prevalence, Incidence, Stimulants, Real-world, Claims database

## Abstract

The objectives of this study were to describe prevalence, incidence, and medications among patients who were diagnosed with narcolepsy in Japan using a claims database. Patients diagnosed with narcolepsy were identified from January 2010 to December 2019 using an employment-based health insurance claims database compiled by JMDC Inc. The prevalence and incidence of narcolepsy were estimated annually in the overall population and by age and sex among employees and their dependents aged < 75 years. Medications, examined for each quarter in the overall population, were modafinil, methylphenidate, pemoline, tricyclic antidepressants, selective serotonin reuptake inhibitors, and serotonin-norepinephrine reuptake inhibitors. We identified 1539 patients with narcolepsy. The overall annual prevalence increased from 5.7 to 18.5/100,000 persons in 2010 and 2019, respectively. Large increases were found from 2010 to 2019 in patients aged 20–29 years and 10–19 years, with the highest prevalence in 2019 (9.7–37.5/100,000 persons and 5.0–27.1/100,000 persons). The overall incidence slightly increased from 3.6 to 4.3/100,000 person-year from 2010 to 2019, and the highest incidence was found in patients aged 20–29 years and 10–19 years (5.8–11.3/100,000 person-year, and 3.8–7.4/100,000 person-year from 2010 to 2019, respectively). Methylphenidate and modafinil were commonly prescribed in 2010 (27.3–38.9% and 17.5–45.5%, respectively). Methylphenidate prescriptions declined during the 10 years, whereas modafinil prescriptions increased (15.6–17.1% and 43.8–45.8% in 2019, respectively). The estimated prevalence and incidence of narcolepsy appeared to increase from 2010 to 2019, especially in teenagers and 20-year olds.

**Supplementary Information:**

The online version contains supplementary material available at 10.1007/s41105-022-00406-4.

## Introduction

Narcolepsy is a chronic neurological disorder characterized by the clinical tetrad of hypersomnia, cataplexy, hypnagogic hallucinations, and sleep paralysis, with additional clinical characteristics of disturbed nocturnal sleep [1]. Narcolepsy has an early onset, typically in adolescence and young adulthood [2, 3]. However, due partly to the under-recognition of its symptoms, the absence of easily measurable biomarkers, and misdiagnosis as other conditions [4], delayed diagnosis (e.g., > 10 years) is common [5]. Narcolepsy negatively affects various aspects of daily life and imposes considerable clinical and economic burden [6–10]. Early diagnosis is therefore essential for individuals affected by this chronic disorder.

Reports on the prevalence and incidence of narcolepsy are scarce, and previous studies in Japan, based on questionnaires and clinical interviews, reported the prevalence of narcolepsy including in teens and young adults was 0.16–0.59% [11–14]. In other parts of Asia, the prevalence among Hong Kong Chinese aged 18–65 years based on questionnaires and laboratory and clinical examinations was reported to be 0.034% [15]. Outside Asia, the estimated prevalence from the Finnish Twin Cohort study based on questionnaires and laboratory examinations was 0.026% [16]. A more recent study from the United States using a health care claims database found a prevalence of 0.0794% (79.4/100 000 persons) among people aged ≤ 65 years [17]. To date, various prevalence estimates have been reported, possibly largely owing to methodological differences (e.g., study designs and disease definitions).

The studies in Japan were conducted a few decades ago. Since then, guidelines for diagnosis and treatment for narcolepsy have been produced [18], and the Japanese Society of Sleep Research has trained a number of specialists and worked continuously to raise awareness of this disorder. However, clarification of how the prevalence of narcolepsy may have changed as a result of these efforts is not available; to the best of our knowledge, little incidence data are available. Thus, the up-to-date nationwide epidemiological data are essential.

Pharmacological treatments are the mainstay for managing narcolepsy. The aforementioned Japanese guidelines for narcolepsy recommend stimulants including modafinil, methylphenidate, and pemoline for treating excessive daytime sleepiness (EDS) [18]. Among these, modafinil is listed as the first-line treatment, as it is a safer option. For treating rapid eye movement (REM) sleep-related symptoms and cataplexy, the guidelines recommend antidepressants including tricyclic antidepressants, selective serotonin reuptake inhibitors (SSRIs), and serotonin-norepinephrine reuptake inhibitors (SNRIs). Although diverse medications are available, to date, the medications indicated for narcolepsy in Japan are those for EDS and tricyclic antidepressants (clomipramine hydrochloride) for REM-related symptoms and cataplexy. As little comparative data on efficacy and safety are available, reports of real-world medication use are also of clinical value.

The objectives of this study were to describe the prevalence, incidence, and medications of patients who were diagnosed with narcolepsy in Japan using a nationwide health insurance claims database from January 2010 to December 2019.

## Materials and methods

### Study design and data source

This descriptive observational study used data from a health insurance claims database compiled by JMDC Inc. (Tokyo, Japan). The data were extracted from January 2009 to December 2019. The JMDC database stores the following anonymized individual-level data: data of people aged < 75 years old who were employed by industries and their dependents (totaling approximately 12 million people [as of July 2021], representing approximately 9.6% of the population in Japan [19]); and individual-level patient and clinical information, including demographics, date-stamped inpatient and outpatient health insurance claims (e.g., diagnosis coded in the International Statistical Classification of Diseases and Related Health Problems, 10th revision [ICD-10], procedures, prescriptions, medical services, costs, and medical institutions), and health check-up records in some individuals.

As the JMDC database includes records of the insured dependents, young individuals in whom narcolepsy onset is typically found (< 30 years old) [2, 3], are included in the database. Additionally, the proportions of teens and young adults aged < 30 years old included in the JMDC database population are higher than those in the general population [20]. Long-term treatment patterns can also be examined with these data because individual medical and treatment history from multiple medical services can be traced, unless an insured employee withdraws from the employment-based health insurance program.

### Ethics statement

As the study used pre-existing, anonymized data whose identifiable personal information could not be reconstructed, it was outside the scope of the Japanese Ethical Guidelines for Medical and Health Research Involving Human Subjects, and ethics approval from an institutional review board was not required. No informed consent was obtained from the individuals included in this study, as all personal and site information were anonymized by the data provider (JMDC Inc.).

### Patient selection

We examined patient data from January 2010 to December 2019 (study period). Patients were included when they met all of the following criteria: (1) records of narcolepsy in 2 data forms (disease name description of “narcolepsy” as well as diagnosis record of narcolepsy in ICD-10 code [G474]) during the study period; (2) diagnosis records of narcolepsy in ≥ 2 consecutive months during the study period (the date of the first diagnosis was defined as index date); and (3) data records available for ≥ 1 year before index date. To estimate incidence for each year, patients were excluded if they had met all of the above three inclusion criteria in previous years; only newly diagnosed individuals were included.

### Outcome measures

The prevalence of narcolepsy, expressed as cases per 100,000 persons, was estimated annually from 2010 to 2019 in the overall population and stratified by age (0–9, 10–19, 20–29, 30–39, 40–49, 50–59, and ≥ 60 years), sex, and their combination. Prevalence was estimated as the number of people who were diagnosed with narcolepsy in one-year period/the total number of people who were included in the database during the same year period. When two consecutive diagnosis records spanned across two years, this diagnosis set was counted in the previous year.

The incidence of narcolepsy, expressed as cases per 100,000 person-year, was estimated annually in the same 10-year period in the overall population and stratified by age, sex, and their combination as for prevalence. Incidence was estimated as the number of people who were newly diagnosed with narcolepsy in a 1-year period/the total number of person time who were at risk of being diagnosed with narcolepsy and were included in the database during the same year period.

Medications examined were modafinil, methylphenidate, pemoline, tricyclic antidepressants (clomipramine and imipramine), SSRIs (paroxetine, fluvoxamine, and trazodone), and SNRIs (milnacipran, venlafaxine, and duloxetine), and we calculated proportion of the above medications prescribed quarterly for each year from 2010 to 2019. Each medication prescribed to a patient multiple times within one category (i.e., modafinil, methylphenidate, pemoline, tricyclic antidepressants, SSRIs, and SNRIs) in a given quarter was counted as one, but one patient could be counted multiple times in the aforementioned 6 categories (i.e., ≥ 1 counts).

### Baseline variables

Baseline patient characteristics assessed at index date were age and sex. Diagnostic tests (polysomnography and multiple sleep latency test [MSLT]) and medications as in the aforementioned outcome measures were assessed from January 2010 to December 2019. The follow-up years were estimated from the date of the first to the last records identified in the database or the end of the study period, whichever occurred first for each patient.

### Statistical analysis

Baseline patient characteristics and outcome measures were summarized descriptively with the mean ± standard deviation (SD) or median (first quartile [Q1], third quartile [Q3]) for continuous variables, and number and percentage for categorical variables. Sensitivity analyses were conducted for prevalence and incidence of narcolepsy using the more strict definitions (the definition described in “Patient selection” plus the following definitions): (1) MSLTs performed in the month of index date and the prior 2 months (in total 3 months); (2) polysomnography tests or MSLTs performed within 2 months before and after index date (in total 5 months); (3) modafinil, methylphenidate, or pemoline prescribed in the month of index date and the following month (in total 2 months); and (4) modafinil, methylphenidate, or pemoline prescribed within 1 months before and after index date (in total 3 months). For (1), the analysis was performed in the overall population and with stratification by sex. Missing data were not imputed. All statistical analyses were performed using SAS Release9.4 (SAS Institute, Inc., NC, USA) or Python 3.8.

## Results

### Patient disposition and baseline characteristics

**Fig. 1:**
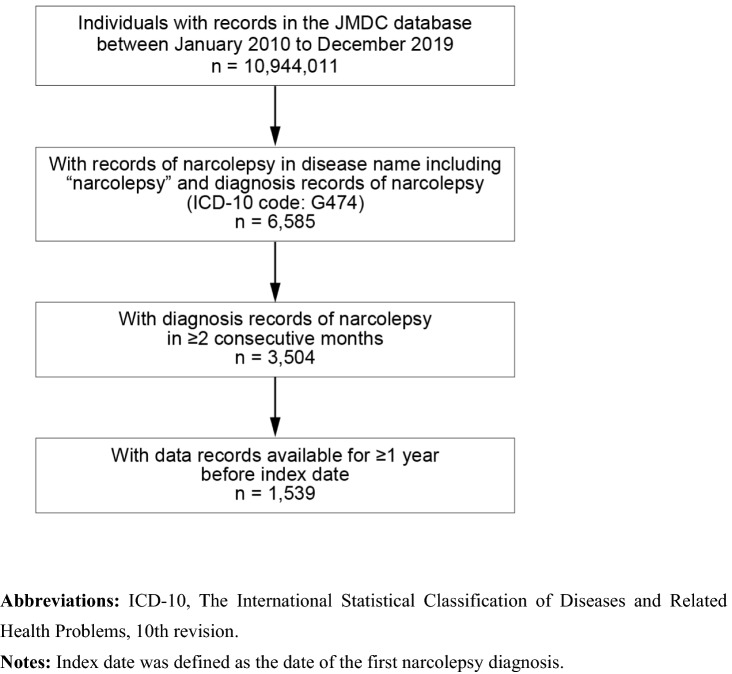
Patient flow. ICD-10, The International Statistical Classification of Diseases and Related Health Problems, 10th revision. Notes: Index date was defined as the date of the first narcolepsy diagnosis

In total, 10,944,011 individuals were identified in the database (Fig. 1). Of those, 6,585 people had records of narcolepsy, and 3,504 people had a narcolepsy diagnosis in ≥ 2 consecutive months. Among those, 1,539 people who had ≥ 1 year of data records before the index date comprised the analysis population.

The baseline patient characteristics are summarized in Table 1. Males represented 62.8% of the population, and the mean ± SD age was 31.51 ± 13.47 years old. Patients aged 10–19 and 20–29 years old represented 22.0% and 30.5% of the total narcolepsy population. Polysomnography and MSLTs were performed in 35.2% and 37.0% of patients. Modafinil was prescribed to 58.3% of patients at baseline, followed by pemoline (25.9%) and methylphenidate (20.2%). The median (Q1, Q3) follow-up time was 2.00 (0.08, 10.01) years.

**Table 1:** Patient characteristics

	Analysis population (*n* = 1539)
	*n* (%)
Sex ^a^, male	966 (62.8)
Age ^a^, years, mean ± SD	31.51 ± 13.47
0–9	11 (0.7)
10–19	339 (22.0)
20–29	470 (30.5)
30–39	255 (16.6)
40–49	275 (17.9)
50–59	164 (10.7)
≥ 60	25 (1.6)
Follow-up time ^b^, years, median (Q1, Q3)	2.00 (0.08, 10.01)
Diagnostic tests ^a^	
Polysomnography, yes	542 (35.2)
Multiple sleep latency test, yes	570 (37.0)
Drugs for narcolepsy ^a^, yes	1539 (100)
Modafinil	898 (58.3)
Methylphenidate	311 (20.2)
Pemoline	399 (25.9)
Tricyclic antidepressants (clomipramine and imipramine)	145 (9.4)
Selective serotonin reuptake inhibitor (paroxetine, fluvoxamine, and trazodone)	308 (20.0)
Serotonin-norepinephrine reuptake inhibitor (milnacipran, venlafaxine, and duloxetine)	340 (22.1)

*SD* standard deviation, *Q1* first quartile, *Q3* third quartile

Notes: Data are expressed as n (%) unless otherwise specified

^a^Sex and age were identified at the date of the first narcolepsy diagnosis (defined as index date), and diagnostic tests and medications for narcolepsy were identified from January 2010 to December 2019. Some patients were prescribed multiple drugs

^b^Follow-up years were from the date of the first to the last records identified in the database or the end of the study period, whichever occurred first

### Prevalence

**Fig. 2:**
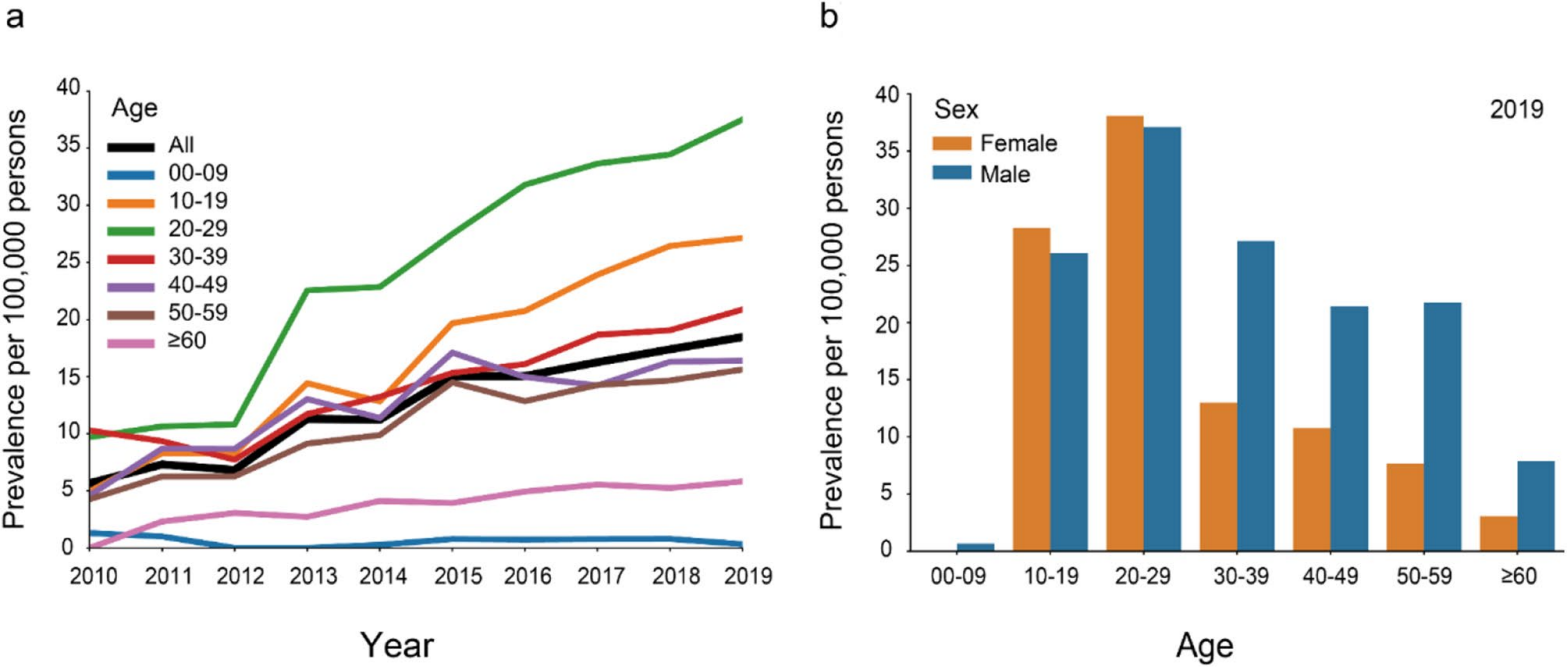
Prevalence of narcolepsy **a** in the overall population and stratified by age from 2010 to 2019 and **b** by age and sex in 2019

The overall prevalence of narcolepsy was 5.7/100,000 persons in 2010, and the prevalence gradually increased to 18.5/100,000 persons in 2019 (Fig. 2a; Table 2). Similarly increasing patterns were observed in all sensitivity analyses (Supplementary files 1 and 2). Age stratification showed similar patterns in most age categories except 0–9 years and ≥ 60 years during the same 10-year period (Fig. 2a). The increase was generally the highest in people aged 20–29 years, followed by those aged 10–19 years, with the highest prevalence in 2019 (9.7–37.5/100,000 persons and 5.0–27.1/100,000 persons, respectively). Prevalence in males were higher than in females throughout the 10-year period, with 21.7/100,000 persons and 14.5/100,000 persons, respectively in 2019 (Table 2). Sex differences in the prevalence in those aged 10–29 years were small, whereas the percentages in males were higher in ages ≥ 30 years than those in females (Fig. 2b).

**Table 2:** Prevalence of narcolepsy from 2010 to 2019 stratified by sex and in the overall population

Year	Male			Female			Total		
	Prevalence per 100,000 persons	Cases	Population	Prevalence per 100,000 persons	Cases	Population	Prevalence per 100,000 persons	Cases	Population
2010	6.2	19	304,758	4.9	12	242,449	5.7	31	547,207
2011	8.4	34	405,545	6.0	20	333,071	7.3	54	738,616
2012	8.0	69	859,198	5.3	35	663,387	6.8	104	1,522,585
2013	13.4	128	954,155	8.6	63	735,614	11.3	191	1,689,769
2014	13.4	195	1,451,139	8.4	96	1,139,320	11.2	291	2,590,459
2015	17.5	267	1,527,217	11.9	143	1,197,396	15.0	410	2,724,613
2016	17.6	385	2,192,007	11.9	207	1,740,756	15.1	592	3,932,763
2017	18.9	509	2,698,060	13.0	277	2,134,536	16.3	786	4,832,596
2018	20.6	665	3,225,166	13.5	355	2,638,278	17.4	1,020	5,863,444
2019	21.7	832	3,828,586	14.5	461	3,171,624	18.5	1,293	7,000,210

### Incidence

**Fig. 3:**
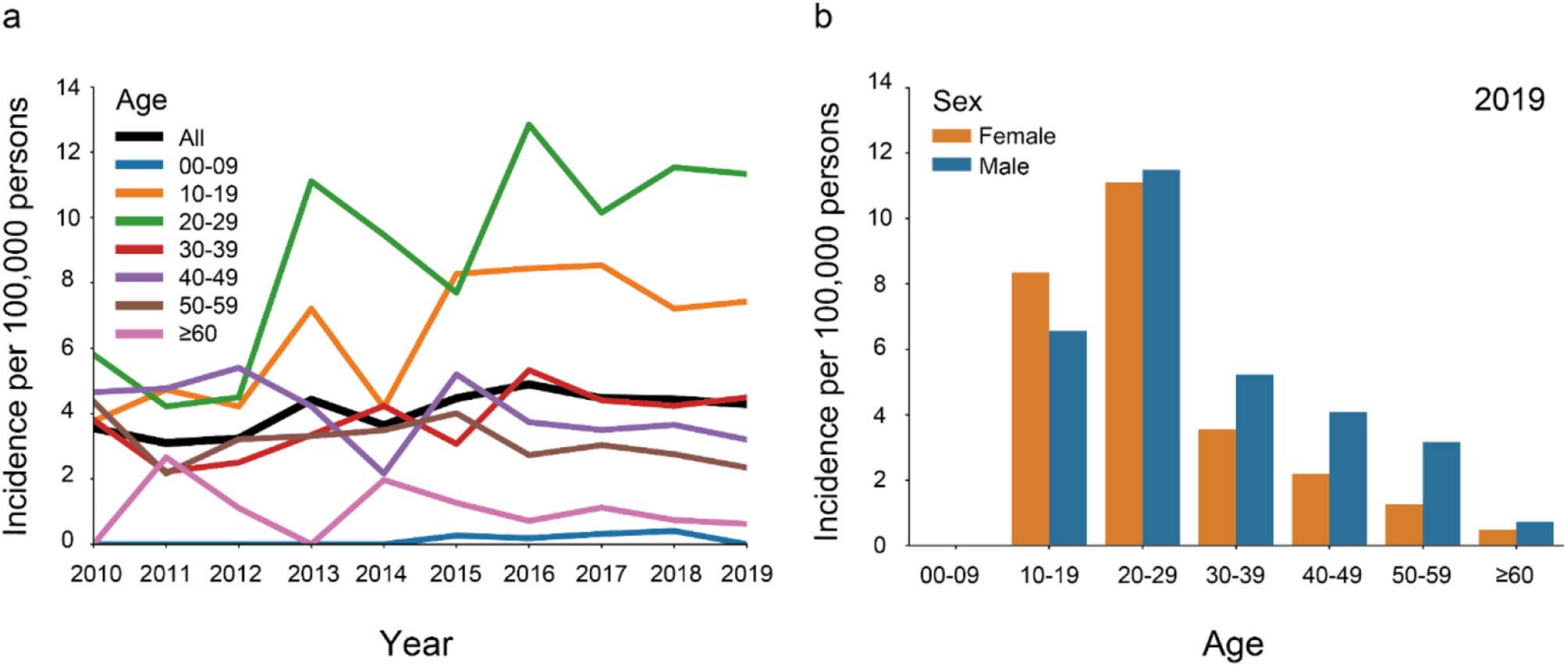
Incidence of narcolepsy **a** in the overall population and stratified by age from 2010 to 2019 and **b** by age and sex in 2019

The overall incidence of narcolepsy slightly increased from 3.6 to 4.3/100,000 person-year in 2010 and 2019 (Fig. 3a; Table 3). All sensitivity analyses showed slight increases during the 10 years (Supplementary files 3 and 4). The highest incidence was found in patients aged 20–29 years and 10–19 years (5.8–11.3/100,000 person-year and 3.8–7.4/100,000 person-year from 2010 to 2019, respectively) (Fig. 3a). For the other age categories, the incidence changed little during 10-year period. Incidence in males and females also changed little during 10 years, but again the incidence in males was mostly higher than females throughout this time period, with the incidence of 4.7/100,000 and 3.8/100,000 person-year, respectively in 2019 (Table 3). Sex differences in the incidence among patients aged 20–29 years were small, but incidence was higher in males than females in the older age categories, whereas in ages 10–19 years, incidence was higher in females than in males in 2019 (Fig. 3b).

**Table 3:** Incidence of narcolepsy from 2010 to 2019 stratified by sex and in the overall population

Year	Male			Female			Total		
	Incidence per 100,000 person-year	Cases	Person time, day	Incidence per 100,000 person-year	Cases	Person time, day	Incidence per 100,000 person-year	Cases	Person time, day
2010	3.4	10	108,899,595	3.8	9	86,119,485	3.6	19	195,019,080
2011	3.8	15	142,951,913	2.2	7	116,067,793	3.1	22	259,019,706
2012	4.2	35	306,379,943	2.0	13	234,597,887	3.2	48	540,977,830
2013	5.1	47	336,787,797	3.5	25	257,073,630	4.4	72	593,861,427
2014	4.5	63	512,727,458	2.6	28	398,931,850	3.6	91	911,659,308
2015	5.0	73	537,751,144	3.9	44	416,799,086	4.5	117	954,550,230
2016	5.4	116	779,675,446	4.2	71	612,795,456	4.9	187	1,392,470,902
2017	4.8	126	952,623,275	4.0	82	744,698,453	4.5	208	1,697,321,728
2018	4.9	152	1,123,580,706	3.8	95	908,066,883	4.4	247	2,031,647,589
2019	4.7	174	1,346,431,384	3.8	114	1,101,607,520	4.3	288	2,448,038,904

### Medications

**Fig. 4:**
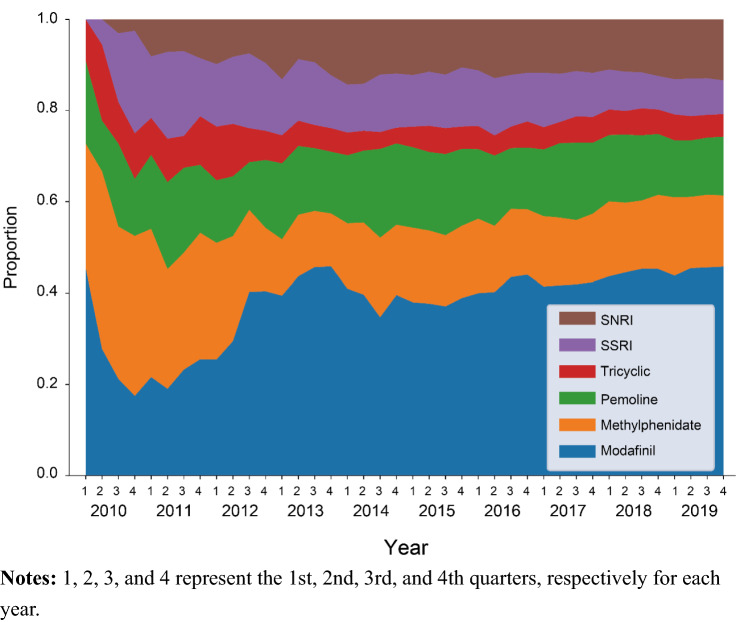
Medications prescribed to patients with narcolepsy from 2010 to 2019 in the overall population. Notes: 1, 2, 3, and 4 represent the 1st, 2nd, 3rd, and 4th quarters, respectively for each year

While methylphenidate was the most commonly prescribed medication in 2010 (27.3–38.9%), the prescriptions declined during 10 years (15.6–17.1% in 2019) (Fig. 4). Modafinil was the second most commonly prescribed medication in 2010 (17.5–45.5%), and prescriptions increased during this period, with the highest prescriptions observed in 2019 (43.8–45.8%). Pemoline was prescribed to 11.1%–18.2% of patients in 2010, and the percentage changed little during 10 years (12.4–12.9% in 2019). Tricyclic antidepressants and SSRIs were prescribed to 9.1–16.7% and 0.0–22.5%, respectively in 2010, and these percentages changed little during this time period (4.9–5.7% and 7.4–8.3%, respectively). In contrast, prescription of SNRIs increased from 0.0 to 3.0% in 2010 to 12.9–13.4% in 2019. The number of patients prescribed modafinil, methylphenidate, pemoline, and other medications was provided in Supplementary file 5.

## Discussion

This study described the prevalence, incidence, and medications of patients diagnosed with narcolepsy using a large-scale health insurance claims data from 2010 to 2019. This study is one of the first to report the up-to-date prevalence, incidence, and real-world medication use among patients with narcolepsy in Japan. The prevalence of narcolepsy appeared to gradually increase over the 10-year period, and although the overall incidence changed little, the incidence in patients in their teens and twenties increased after around 2013. Methylphenidate was most commonly prescribed in 2010, but its prescription slightly declined thereafter. Conversely, modafinil was the second most commonly prescribed medication in 2010, and its use increased thereafter to 43.8–45.8% in 2019.

### Prevalence and incidence

We found the estimated prevalence was higher in people aged 10–29 years than in other age groups, and this finding conforms to the general understanding of narcolepsy onset [2, 3]. Although a direct comparison with previous prevalence estimates is not meaningful due to differences in study designs, disease definitions, and time periods [11, 12], our results were generally lower than those previously reported in both adults and teens/twenties. The overall prevalence in our study in 2019 was 0.0185% (18.5/100,000 persons), whereas previously reported prevalence rates estimated using questionnaires and interviews were 0.16–0.59% [11, 12]. The prevalence in patients in their teens and twenties in our study was 0.0271% (27.1/100,000 persons) and 0.0375% (37.5/100,000 persons), respectively. Again, our estimates were lower than those reported previously (0.71% and 0.75% in teens and twenties, respectively) [12]. Additionally, our estimates were lower than those reported in a study in United States using healthcare claims data (79.4/100 000 persons aged less than 66 years) [17].

Such seemingly low prevalence in our study may suggest that there may be people with narcolepsy in the general population but who have not visited medical institutions. Presumably, access to hospitals/clinics where diagnostic tests can be performed is limited, and it is possible that patients may have been treated in near-by hospitals/clinics, by attention-deficit/hyperactivity disorder (ADHD) being recorded. Additionally, ADHD is more recognized among young people, and it is possible that narcolepsy may have been misdiagnosed with ADHD in these people. Narcolepsy is a lifelong disorder without a known cure. It was previously found that after people are diagnosed with narcolepsy, healthcare utilization and overall medical costs decreased [21]. Prompt diagnosis is therefore important to reduce clinical and economic burden of narcolepsy.

The comparison of our results with those of previous studies overseas is not straightforward because of various methodologies use and time periods. Previously reported prevalence estimates were 0.034% among Hong Kong Chinese [15], 0.026% in the Finnish Twin Cohort [16] using questionnaires and clinical and laboratory examinations, and 0.0794% (79.4/100 000 persons) in a study in the United States using healthcare claims data [17]. As for the incidence of narcolepsy, which has been further sporadically reported previously, methodologies and years differed among studies. Nonetheless, based on our definitions using polysomnography or MSLT claims, our annual incidence rates were 0.4–2.2/100 000 persons from 2010 to 2019 (Supplementary file 4). The overall annual incidence from the above study in the United States using health care claims data was approximately 5–8/100 000 persons based on the 3 definitions using MSLT claims records (e.g., patients with a polysomnography claim and MSLT claims ± 180 days before the first narcolepsy diagnosis, using longer definitions than our study) [17]. Additionally, based on chart reviews using a study’s own diagnostic criteria as well as laboratory and clinical features, another study reported an annual incidence rate of 1.37/100 000 persons between 1960 and 1989 [22].

We observed an overall increase in the prevalence during 10-year period, and the prevalence and incidence estimates in individuals aged 10–29 years increased during this period. These increasing patterns were also confirmed by sensitivity analyses using four definitions for narcolepsy. The increase we found in this study may be due to growing awareness of this illness among doctors and general populations. With the spread of the Internet and mobile phones/tablets, teens are now able to research symptoms without assistance from adults, and parents are also able to research and gain knowledge regarding their children's symptoms including daytime napping. Additionally, the number of outpatient sleep clinics has increased recently, possibly facilitating visits to these clinics over visits to psychiatrists Additionally, as aforementioned, the Japanese Society of Sleep Research has trained specialists and worked continuously to raise awareness of narcolepsy. It is possible that these recent changes and efforts might have partially contributed to diagnosis in young individuals.

### Medications

SNRIs were prescribed to only 0.0–3.0% of patients in 2010, the year duloxetine was marketed (April 2010), and its use increased thereafter (approximately 13% in 2019). Conversely, prescriptions of tricyclic antidepressants and SSRIs changed little over the 10-year period, while larger changes were observed in methylphenidate and modafinil. Methylphenidate was most commonly prescribed in 2010, but those prescriptions decreased across the 10 years. This decreasing rate is supposedly because, since 2007, methylphenidate has been only prescribed by registered methylphenidate physicians and pharmacists in Japan [23, 24]. Recent guidelines in Europe and the United States list methylphenidate as the second-line therapy for treating EDS [25, 26], and the latter guidelines describe this drug as a second-line option when other stimulants show no effectiveness.

Modafinil use surpassed methylphenidate in later years, and approximately half of patients with narcolepsy were prescribed modafinil in 2019. This common use of modafinil corresponds to the treatment guidelines in Japan, which list modafinil as the first-line treatment for EDS due to potential dependency of methylphenidate [18]. European and American guidelines also recommend modafinil as the first-line therapy, along with pitolisant, sodium oxybate, solriamfetol [25], and armodafinil [26]; the latter four medications are not available in Japan. Generally accepted low abuse potential of modafinil [27] may be related to its common use in Japan.

Pemoline has been used to a lesser degree since 2010, but it was constantly used by approximately 10%–20% of patients during the 10-year period. This result corresponds to pemoline being one of commonly used medication in clinical practice in Japan [28]. Its characteristics (e.g., relatively long half-life [8–10 h] [28]) and patients who cannot use other drugs due to including side effects might be related to this result. Although pemoline is indicated for narcolepsy in Japan, it is not listed in the guidelines in Europe and the Unites States [25, 26], and is not available in the latter country due to its potential lethal hepatotoxicity [29]. Due to safety concern, the Japanese guidelines recommend liver function monitoring during treatment [18]. Additionally, it is possible that our study included patients with other disorders (e.g., idiopathic hypersomnia) to whom pemoline might have been prescribed.

### Limitations

The findings of this study may not be generalizable to the entire population with narcolepsy in Japan, and are interpreted within the context of the following limitations. First, we used an employment-based health insurance claims data collected from employees and their dependents of relatively large corporates. The size of the corporations and occupations/industries were however unknown because this information was not disclosed by the data provider for the protection of personal information. Not many people aged 65 years or older were included in the database, and people aged 75 years or older were not included; however, children and adolescents, who may develop narcolepsy, are included [20], enabling us to examine pediatric narcolepsy in a large study population. Second, diagnosis records of narcolepsy extracted from the health insurance claims database were entered for billing purposes and, as aforementioned, may not reflect actual diagnoses, which may have influenced estimates of prevalence and incidence and medication use. There may have been patients with narcolepsy who were prescribed methylphenidate but whose recorded diagnosis differed from narcolepsy (e.g., ADHD), and who therefore could not be captured in this study. Conversely, the prevalence and incidence estimates may have included patients with other disorders. Presumably, modafinil was prescribed to patients with idiopathic hypersomnia in clinical practice under narcolepsy disease codes until March 2020 (later modafinil’s indication was expanded to this disorder). Third, as described previously, individuals’ health insurance data are recorded from the time they joined the insurance plan and continued as long as they were covered. Their clinical and treatment history before joining an insurance were therefore not traceable. The included individuals may have been diagnosed with narcolepsy before they (and their dependents) joined the insurance plan. Lastly, as subtypes of narcolepsy (i.e., narcolepsy type 1 and 2 [30]) were not recorded in the database, and prescribed medications were examined in the overall population.

## Conclusions

This study described up-to-date epidemiological data and medications among patients with narcolepsy from 2010 to 2019 in a Japanese real-world setting. The estimated prevalence of narcolepsy appeared to increase during the 10-year period, and new cases of narcolepsy were diagnosed at higher rates in individuals in their teens and twenties. Further research including comorbidities and healthcare resource utilization is of clinical value.

### Supplementary Information

Below is the link to the electronic supplementary material.

Supplementary file1 (PDF 327 KB)

## Data Availability

The data that support the findings of this study are available for purchase from JMDC Inc. Restrictions apply to the availability of the data used in this study because of contractual agreements between JMDC Inc. and health insurance associations. For inquiries about accessibility to the dataset, please contact JMDC Inc. (Website, https://www.jmdc.co.jp/en/index; E-mail: mdbhelp@jmdc.co.jp).

## References

[1] Fromherz S, Mignot E (2004). Narcolepsy research: past, present, and future perspectives. Arch Ital Biol.

[2] Longstreth WT, Koepsell TD, Ton TG (2007). The epidemiology of narcolepsy. Sleep.

[3] Barker EC, Flygare J, Paruthi S (2020). Living with narcolepsy: Current management strategies, future prospects, and overlooked real-life concerns. Nat Sci Sleep.

[4] Kryger MH, Walid R, Manfreda J (2002). Diagnoses received by narcolepsy patients in the year prior to diagnosis by a sleep specialist. Sleep.

[5] Thorpy MJ, Hiller G (2017). The medical and economic burden of narcolepsy: Implications for managed care. Am Health Drug Benefits.

[6] Carls G, Reddy SR, Broder MS (2020). Burden of disease in pediatric narcolepsy: a claims-based analysis of health care utilization, costs, and comorbidities. Sleep Med.

[7] Black J, Reaven NL, Funk SE (2017). Medical comorbidity in narcolepsy: findings from the Burden of Narcolepsy Disease (BOND) study. Sleep Med.

[8] Dodel R, Peter H, Spottke A (2007). Health-related quality of life in patients with narcolepsy. Sleep Med.

[9] Flores NM, Villa KF, Black J (2016). The humanistic and economic burden of narcolepsy. J Clin Sleep Med.

[10] Ohayon MM, Black J, Lai C (2014). Increased mortality in narcolepsy. Sleep.

[11] Honda Y, Takahashi Y. Diagnosis of narcolepsy and its treatments: A survey of adolescent sleep and lifestyle and narcolepsy prevalence. Report of the research funded by the Ministry of Health and Welfare of Japan. 1998:330 [in Japanese].

[12] Kambayashi T, Tashiro T, Iijima S (1992). An epidemiological study on prevalence of narcolepsy in Japanese. Jpn J Psychiatry Neurol.

[13] Honda Y (1979). Census of narcolepsy, cataplexy, and sleep life among teen-agers in Fujisawa City (abstract). Sleep Res.

[14] Tashiro T, Kanbayashi T, Iijima S (1992). An epidemiological study of narcolepsy in Japanese (abstract). J Sleep Res.

[15] Wing YK, Li RH, Lam CW (2002). The prevalence of narcolepsy among Chinese in Hong Kong. Ann Neurol.

[16] Hublin C, Partinen M, Kaprio J (1994). Epidemiology of narcolepsy. Sleep.

[17] Scheer D, Schwartz SW, Parr M (2019). Prevalence and incidence of narcolepsy in a US health care claims database, 2008–2010. Sleep.

[18] Japanese Society of Sleep Research. Guidelines of diagnosis and treatment for narcolepsy, p.1–22. https://jssr.jp/files/guideline/narcolepsy.pdf [in Japanese]. Accessed 16 Feb 2022.

[19] Ministry of Internal Affairs and Communications. Population estimates – July 2021. 2021. https://www.stat.go.jp/data/jinsui/pdf/202107.pdf [in Japanese]. Accessed 16 Feb 2022.

[20] Hamada K, Aoki K (2012). 5. Introduction of database application examples – Receipt from the standpoint of pharmaceutical companies (JMDC) – An example of analysis of cancer incidence trends and disease trends after anticancer drug administration. Jpn J Pharmacoepidemiol..

[21] Villa KF, Reaven NL, Funk SE (2018). Changes in medical services and drug utilization and associated costs after narcolepsy diagnosis in the United States. Am Health Drug Benefits.

[22] Silber MH, Krahn LE, Olson EJ (2002). The epidemiology of narcolepsy in Olmsted County, Minnesota: a population-based study. Sleep.

[23] Inoue Y, Sakuta K (2010). Diagnosis and treatment of sleeping disorder: with a focus on narcolepsy. Farumashia.

[24] Pharmaceuticals and Medical Devices Agency. Ritanin tablets 10mg (methylphenidate). Package insert. 2021. https://www.info.pmda.go.jp/go/pack/1179009F1035_1_05/ [in Japanese] Accessed 16 Feb 2022.

[25] Bassetti CLA, Kallweit U, Vignatelli L (2021). European guideline and expert statements on the management of narcolepsy in adults and children. Eur J Neurol.

[26] Franceschini C, Pizza F, Cavalli F (2021). A practical guide to the pharmacological and behavioral therapy of Narcolepsy. Neurotherapeutics.

[27] Myrick H, Malcolm R, Taylor B (2004). Modafinil: preclinical, clinical, and post-marketing surveillance-A review of abuse liability issues. Ann Clin Psychiatry.

[28] Honda M (2007). Treatment for sleeping disorder. Folia Pharmacologica Japonica.

[29] Morgenthaler TI, Kapur VK, Brown T (2007). Practice parameters for the treatment of narcolepsy and other hypersomnias of central origin. Sleep.

[30] Sateia MJ (2014). International classification of sleep disorders-third edition: Highlights and modifications. Chest.

