# Correction to: Prevalence, incidence, and medications of narcolepsy in Japan: a descriptive observational study using a health insurance claims database

**DOI:** 10.1007/s41105-022-00427-z

**Published:** 2022-10-16

**Authors:** Aya Imanishi, Yuta Kamada, Kai Shibata, Yukinori Sakata, Hiroaki Munakata, Mika Ishii

**Affiliations:** 1 Department of Neuropsychiatry, Akita University School of Medicine, Akita, Japan; 2 Clinical Planning and Development Department, Medical HQs, Eisai Co., Ltd, Tokyo, Japan

## Correction to: Sleep and Biological Rhythms (2022) 20:585-594 10.1007/s41105-022-00406-4

The article “Prevalence, incidence, and medications of narcolepsy in Japan: a descriptive observational study using a health insurance claims database”, written by Aya Imanishi, Yuta Kamada, Kai Shibata, Yukinori Sakata, Hiroaki Munakata, Mika Ishii, was originally published electronically on the publisher’s internet portal on 30 August 2022 without open access. With the author(s)’ decision to opt for Open Choice the copyright of the article changed on 01 October 2022 to © The Authors 2022 and the article is forthwith distributed under a Creative Commons Attribution 4.0 International License, which permits use, sharing, adaptation, distribution and reproduction in any medium or format, as long as you give appropriate credit to the original author(s) and the source, provide a link to the Creative Commons licence, and indicate if changes were made. The images or other third party material in this article are included in the article's Creative Commons licence, unless indicated otherwise in a credit line to the material. If material is not included in the article's Creative Commons licence and your intended use is not permitted by statutory regulation or exceeds the permitted use, you will need to obtain permission directly from the copyright holder. To view a copy of this licence, visit http://creativecommons.org/licenses/by/4.0/.

