# Supplementary material for: Prevalence, incidence, and medications of narcolepsy in Japan: a descriptive observational study using a health insurance claims database
Source: Sleep Biol Rhythms. 2022 Aug 30;20(4):585–94. doi: 10.1007/s41105-022-00406-4 (PMC10899966; doi:10.1007/s41105-022-00406-4)
Supplement: Supplementary file 1 — Supplementary file1 (PDF 327 KB) [file 41105_2022_406_MOESM1_ESM.pdf]

Supplementary materials

Article Title:
Prevalence, incidence, and medications of narcolepsy in Japan: A descriptive observational study using a health insurance claims database

Journal name:
Sleep and Biological Rhythms

Author names:
Aya Imanishi, Yuta Kamada, Kai Shibata, Yukinori Sakata, Hiroaki Munakata, Mika Ishii

Affiliation and e-mail address of the corresponding author:
Aya Imanishi
[1] Department of Neuropsychiatry, Akita University School of Medicine, Akita, Japan
1-1-1 Hondo, Akita city, Akita, 010-8543, Japan
Tel: +81 18 884 6122; Fax: +81 18 884 6322
E-mail: ima24@med.akita-u.ac.jp

Supplementary file 1. Prevalence of narcolepsy from 2010 to 2019 stratified by sex and in the overall population using a definition with multiple sleep latency tests for narcolepsy.

Year	Sensitivity analysis 1 [a]								
	**Male**			**Female**			**Total**		
	**Prevalence per 100,000 persons**	**Cases**	**Population**	**Prevalence per 100,000 persons**	**Cases**	**Population**	**Prevalence per 100,000 persons**	**Cases**	**Population**
2010	0.7	2	304,758	0.0	0	242,449	0.4	2	547,207
2011	1.2	5	405,545	0.3	1	333,071	0.8	6	738,616
2012	1.6	14	859,198	0.9	6	663,387	1.3	20	1,522,585
2013	4.2	40	954,155	1.2	9	735,614	2.9	49	1,689,769
2014	4.0	58	1,451,139	1.4	16	1,139,320	2.9	74	2,590,459
2015	4.5	68	1,527,217	1.8	21	1,197,396	3.3	89	2,724,613
2016	4.3	95	2,192,007	2.2	39	1,740,756	3.4	134	3,932,763
2017	4.7	127	2,698,060	2.7	57	2,134,536	3.8	184	4,832,596
2018	5.2	169	3,225,166	3.1	83	2,638,278	4.3	252	5,863,444
2019	5.6	215	3,828,586	3.4	109	3,171,624	4.6	324	7,000,210

Notes:

[a] Narcolepsy was defined using the definition described in "Patient selection", plus multiple sleep latency tests performed in the month of index date and the prior 2 months (in total 3 months).

Supplementary file 2. Overall prevalence of narcolepsy from 2010 to 2019 using three definitions for narcolepsy.

	Sensitivity analysis 2 [a]			Sensitivity analysis 3 [b]			Sensitivity analysis 4 [c]		
**Year**	**Prevalence per 100,000**	**Cases**	**Population**	**Prevalence per 100,000**	**Cases**	**Population**	**Prevalence per 100,000**	**Cases**	**Population**
2010	0.9	5	547,207	4.9	27	547,207	4.9	27	547,207
2011	2.0	15	738,616	6.0	44	738,616	6.0	44	738,616
2012	2.8	43	1,522,585	5.2	79	1,522,585	5.2	79	1,522,585
2013	5.1	87	1,689,769	8.8	149	1,689,769	8.8	149	1,689,769
2014	4.9	126	2,590,459	8.8	229	2,590,459	8.8	229	2,590,459
2015	5.7	155	2,724,613	11.6	317	2,724,613	11.7	318	2,724,613
2016	6.0	235	3,932,763	11.4	450	3,932,763	11.5	451	3,932,763
2017	6.4	307	4,832,596	12.5	603	4,832,596	12.5	606	4,832,596
2018	7.1	416	5,863,444	13.3	781	5,863,444	13.4	784	5,863,444
2019	7.9	553	7,000,210	14.3	1,003	7,000,210	14.4	1,006	7,000,210

Notes:

Narcolepsy was defined using the definition described in "Patient selection" plus the following additional definitions:

[a] Definitions of narcolepsy defined as polysomnography tests or multiple sleep latency tests performed within 2 months before and after index date (in total 5 months).

[b] Definitions of narcolepsy defined as modafinil, methylphenidate, or pemoline prescribed in the month of index date (the month of the first narcolepsy diagnosis of ≥2 consecutive diagnosis in separate months) and the following one month (in total 2 months).

[c] Definitions of narcolepsy defined as modafinil, methylphenidate, or pemoline prescribed within 1 months before and after index date (in total 3 months).

Supplementary file 3. Incidence of narcolepsy from 2010 to 2019 stratified by sex and in the overall population using a definition with multiple sleep latency tests for narcolepsy.

Year	Sensitivity analysis 1 [a]								
	**Male**			**Female**			**Total**		
	**Incidence per 100,000 person-year**	**Cases**	**Person time, day**	**Incidence per 100,000 person-year**	**Cases**	**Person time, day**	**Incidence per 100,000 person-year**	**Cases**	**Person time, day**
2010	0.0	0	108,901,179	0.0	0	86,121,276	0.0	0	195,022,455
2011	0.8	3	142,957,021	0.3	1	116,071,350	0.6	4	259,028,371
2012	1.1	9	306,391,763	0.3	2	234,604,561	0.7	11	540,996,324
2013	2.1	19	336,807,869	0.4	3	257,085,157	1.4	22	593,893,026
2014	1.3	18	512,758,452	0.5	6	398,950,282	1.0	24	911,708,734
2015	1.0	14	537,799,680	0.3	3	416,827,067	0.6	17	954,626,747
2016	1.1	24	779,743,786	1.1	19	612,835,080	1.1	43	1,392,578,866
2017	1.3	35	952,720,135	1.0	20	744,751,973	1.2	55	1,697,472,108
2018	1.4	43	1,123,705,040	1.1	27	908,136,277	1.3	70	2,031,841,317
2019	1.4	52	1,346,585,089	1.0	30	1,101,694,719	1.2	82	2,448,279,808

Notes:

[a] Narcolepsy was defined using the definition described in "Patient selection", plus multiple sleep latency tests performed in the month of index date and the prior 2 months (in total 3 months).

Supplementary file 4. Overall incidence of narcolepsy from 2010 to 2019 using three definitions for narcolepsy.

Year	Sensitivity analysis 2 [a]			Sensitivity analysis 3 [b]			Sensitivity analysis 4 [c]		
	**Incidence per 100,000 person-year**	**Cases**	**Person time, day**	**Incidence per 100,000 person-year**	**Cases**	**Person time, day**	**Incidence per 100,000 person-year**	**Cases**	**Person time, day**
2010	0.4	2	195,022,059	3.2	17	195,019,292	3.2	17	195,019,292
2011	1.4	10	259,026,640	2.3	16	259,021,226	2.3	16	259,021,226
2012	1.7	25	540,990,139	2.4	35	540,983,556	2.4	35	540,983,556
2013	2.2	35	593,883,263	3.6	59	593,870,398	3.6	59	593,870,398
2014	1.5	37	911,695,624	3.0	75	911,671,842	3.0	75	911,671,842
2015	1.3	33	954,610,261	3.3	86	954,570,911	3.3	87	954,570,606
2016	2.2	84	1,392,554,847	3.7	140	1,392,502,758	3.7	140	1,392,502,392
2017	1.8	86	1,697,438,776	3.5	163	1,697,366,366	3.5	165	1,697,365,850
2018	2.2	121	2,031,796,460	3.4	188	2,031,708,937	3.4	188	2,031,707,842
2019	2.2	150	2,448,218,405	3.6	242	2,448,114,065	3.6	242	2,448,113,091

Notes:

Narcolepsy was defined using the definition described in "Patient selection" plus the following additional definitions:

[a] Definitions of narcolepsy defined as polysomnography tests or multiple sleep latency tests performed within 2 months before and after index date (in total 5 months).

[b] Definitions of narcolepsy defined as modafinil, methylphenidate, or pemoline prescribed in the month of index date (the month of the first narcolepsy diagnosis of ≥2 consecutive diagnosis in separate months) and the following one month (in total 2 months).

[c] Definitions of narcolepsy defined as modafinil, methylphenidate, or pemoline prescribed within 1 months before and after index date (in total 3 months).

Supplementary file 5. The number of patients prescribed medications for narcolepsy by quarter from 2010 to 2019.

Year_quarter	Modafinil	Methylphenidate	Pemoline	Tricyclic antidepressants [a]	SSRIs [b]	SNRIs [c]
2010_1Q	5	3	2	1	0	0
2010_2Q	5	7	2	3	1	0
2010_3Q	7	11	6	3	5	1
2010_4Q	7	14	5	4	9	1
2011_1Q	8	12	6	3	5	3
2011_2Q	8	11	8	4	8	3
2011_3Q	10	11	8	3	8	3
2011_4Q	12	13	7	5	6	4
2012_1Q	13	13	7	6	7	5
2012_2Q	18	14	8	7	9	5
2012_3Q	27	12	7	5	11	5
2012_4Q	38	13	14	6	14	9
2013_1Q	45	14	19	7	14	15
2013_2Q	55	17	19	7	17	11
2013_3Q	63	17	19	7	19	13
2013_4Q	71	18	21	8	18	19
2014_1Q	66	23	24	8	17	23
2014_2Q	73	29	29	8	19	26
2014_3Q	66	33	37	7	24	23
2014_4Q	80	31	36	7	24	24
2015_1Q	84	36	39	10	25	27
2015_2Q	92	39	42	14	29	28
2015_3Q	98	41	47	15	31	32
2015_4Q	111	45	48	14	37	30
2016_1Q	118	48	45	15	36	33
2016_2Q	128	46	49	14	40	41
2016_3Q	157	54	48	17	41	44
2016_4Q	169	55	52	22	41	45
2017_1Q	170	63	60	20	49	48
2017_2Q	189	67	74	21	48	54
2017_3Q	203	68	82	28	48	55
2017_4Q	210	74	77	28	48	58
2018_1Q	234	88	78	30	47	59
2018_2Q	248	85	83	29	48	64
2018_3Q	269	89	85	35	47	69
2018_4Q	295	106	87	35	48	81
2019_1Q	294	115	84	38	52	88
2019_2Q	308	106	84	36	56	88
2019_3Q	346	121	95	38	61	98
2019_4Q	352	120	99	38	57	103

Abbreviations: SSRI, selective serotonin reuptake inhibitor; SNRI, serotonin-norepinephrine reuptake inhibitor.

Notes: Each medication prescribed to a patient multiple times within one category (modafinil, methylphenidate, pemoline, tricyclic antidepressants, SNRIs, and SNRIs) in a given quarter was counted as one. One patient could be counted multiple times in the aforementioned 6 categories.

[a] Tricyclic antidepressants: clomipramine and imipramine.

[b] SSRIs: paroxetine, fluvoxamine, and trazodone.

[c] SNRIs: milnacipran, venlafaxine, and duloxetine.
